# Diagnostic Accuracy of a Simple and Rapid Diagnostic Test RLDT for the Detection of Cholera in Bangladesh

**DOI:** 10.1093/ofid/ofag292

**Published:** 2026-05-14

**Authors:** Kamrul Islam, Ashraful Islam Khan, M D Taufiqur Rahman Bhuiyan, Fan He, Zahid Hasan Khan, Md Taufiqul Islam, Firdausi Qadri, Subhra Chakraborty

**Affiliations:** International Centre for Diarrhoeal Disease Research, Bangladesh (icddr,b), Dhaka, Bangladesh; International Centre for Diarrhoeal Disease Research, Bangladesh (icddr,b), Dhaka, Bangladesh; International Centre for Diarrhoeal Disease Research, Bangladesh (icddr,b), Dhaka, Bangladesh; Department of International Health, Johns Hopkins Bloomberg School of Public Health, Johns Hopkins University, Baltimore, Maryland, USA; International Centre for Diarrhoeal Disease Research, Bangladesh (icddr,b), Dhaka, Bangladesh; International Centre for Diarrhoeal Disease Research, Bangladesh (icddr,b), Dhaka, Bangladesh; International Centre for Diarrhoeal Disease Research, Bangladesh (icddr,b), Dhaka, Bangladesh; Department of International Health, Johns Hopkins Bloomberg School of Public Health, Johns Hopkins University, Baltimore, Maryland, USA

**Keywords:** cholera, diagnostic test, diarrhea, RDT, RLDT

## Abstract

**Background:**

The rising incidence and case fatality rates of cholera outbreaks underscore the need for better diagnostic tests for early detection and confirmation of the outbreak to enable rapid responses and obtain reliable surveillance data. We evaluated the Rapid LAMP-based Diagnostic Test (RLDT) for detecting cholera to determine if this test has the potential to fill the gap.

**Methods:**

This study included training and evaluation of RLDT in the cholera reference laboratory in Bangladesh. Stool samples collected from suspected cholera patients seeking care at the icddr,b hospital, Dhaka, were tested using cholera RLDT, microbiological culture, Rapid Diagnostic Test (RDT crystal VC O1, O139), and PCR and compared.

**Results:**

Out of 205 cases analyzed, 136 (66%) were positive for cholera by RLDT, 131 (64%) by PCR, 92 (45%) by culture, and 90 (44%) by RDT. The RLDT was highly specific, as all the culture positives were positive by RLDT, except 1 (sensitivity of 99%), and could detect all the PCR positives except 2 (sensitivity of 97%). Almost perfect agreement was found between RLDT and PCR (kappa 0.9). Rapid LAMP-based Diagnostic Test being more sensitive than culture and RDT, the agreement was moderate (kappa 0.6 and 0.5, respectively). Rapid Diagnostic Test missed detection of cholera in 11 samples, positive by other tests. The accuracy of RLDT was 100% in detecting cholera from water samples.

**Conclusions:**

Rapid LAMP-based Diagnostic Test was specific and sensitive, detecting 21% more cholera-positive cases than culture or RDT. Rapid LAMP-based Diagnostic Test has the potential to improve the rapid detection of cholera in outbreak situations and for surveillance data.

## Teaser

Rapid LAMP-based Diagnostic Test could be implemented in a reference laboratory in low and middle-income where cholera is endemic. Rapid LAMP-based Diagnostic Test is highly specific and more sensitive compared to culture and Rapid Diagnostic Test for the detection of cholera. Rapid LAMP-based Diagnostic Test could be scaled up for cholera detection.

Cholera is a severe dehydrating diarrheal disease caused by *Vibrio cholerae* serogroup O1 and sporadically by *V. cholerae* O139. Globally, 3–5 million cases and over 120 000 deaths occur annually due to cholera [1]. Approximately 1.3 billion people are at risk of cholera around the world [2, 3].

Stool culture remains the gold standard for laboratory detection and surveillance of cholera, although stool culture is not 100% sensitive [4, 5]. Culturing stool or rectal swab on the selective media either directly or after enrichment in alkaline peptone water (APW), followed by biochemical analysis, serotyping, and bio-typing with antisera [6–8], takes 2–4 days and requires competent laboratory support and technical skills, which are not always available where cholera is endemic.

Several PCR methods have been developed, but a standard, validated technique is not available [9]. PCR requires trained technicians, a well-equipped laboratory, and a supply of cold chain–maintained reagents that are not often available outside the central laboratories.

Several cholera Rapid Diagnostic Tests (RDTs) have been developed. The commonly used RDTs are based on the lateral flow that detects the lipopolysaccharide of *V. cholerae* O1 and O139 by immunochromatographic assay [10]. Although RDTs are well-suited to meet the demand for cholera diagnosis where culture or PCR-based methods are not feasible, these RDTs suffer from limitations. The most evaluated RDT, Crystal VC (Arkray Healthcare Pvt. Ltd, Gujarat, India), has demonstrated a wide variation of sensitivity and specificity (ranging from 58% to 100% respectively) [4, 11–18]. A systematic review of diagnostic tests for cholera demonstrated a notable absence of evidence to support the use of these rapid tests [11] as they did not meet the expected minimal performance of a sensitivity of at least 90% and a specificity of at least 85% recommended by the Global Task Force for Cholera Control for the Target Product Profile of RDT [19]. Cholera surveillance and disease burden estimation also rely on the accuracy of the diagnostic assay.

The case fatality rate is often highest at the beginning of an outbreak or epidemic, and delayed recognition of a cholera outbreak results in a delayed public health response, resulting in high mortality [4]. Therefore, there is a crucial need for a rapid cholera diagnostic test with better accuracy, which can confirm the outbreak early and at the outbreak site to initiate rapid response and also obtain reliable surveillance data to identify the priority areas for multisectoral intervention.

Cholera is a water-borne infectious disease. Methods for identifying culturable *V. cholerae* from water and sewage include culture and PCR techniques [20–22]. We previously adapted the Crystal VC dipstick assay and developed a simple technique for identifying cholera in water [23]. However, this assay requires incubation in APW for 24 hours. A rapid but sensitive assay would be valuable to quickly identify if water sources are contaminated with cholera.

To address these challenges in the detection of cholera, Chakraborty et al [10] developed a simple, sensitive, and rapid detection test, “Rapid LAMP (Loop-mediated Isothermal Amplification)–based Diagnostic Test (RLDT),” for the detection of cholera from both fecal and water samples, which is cold-chain and almost electricity-free. The RLDT detects cholera directly from stool in less than an hour, with the lowest detection limit similar to quantitative PCR [10]. In this study, we evaluated the performance of the RLDT test in comparison with conventional PCR, culture, and the commercial RDT, Crystal VC, in the reference laboratory of a cholera-endemic country, Bangladesh.

## METHODS

### Ethics Statement

This study was approved by the Research Review and the Ethical Review Committees of the International Centre for Diarrhoeal Disease Research, Bangladesh (icddr,b) and the Institutional Review Board of the Johns Hopkins University. Written informed consent was obtained from the caregiver of the children, assent from children those 11–17 years of age, and adults gave their own consent.

### Study Participants and Specimen Collection

We collected stool specimens from 210 adults and children who were seeking care for diarrhea at the Dhaka Hospital of the icddr,b, Bangladesh, during October 2021 to April 2022. Of note, from March 1 to April 10, one of the worst cholera outbreaks occurred in Dhaka, and a huge number of patients attended the icddr,b hospital for treatment [24]. Thus, we had the opportunity to test the RLDT during a cholera outbreak in this study.

### Diagnostic Tests to Detect Cholera From Stool

Microbiological culture was performed by culturing stool directly on taurocholate-tellurite gelatin agar plates or after enrichment in APW (1% peptone, 1% NaCl; pH 8.5) for overnight, followed by confirmation of the *V. cholerae* isolates by slide agglutination with monoclonal antibodies specific to *V*. *cholerae* serovar O1 (Ogawa or Inaba) and O139.

The Crystal VC RDT (includes both O1 and O139) was performed on fresh stool samples according to the manufacturer's instructions. In brief, liquid stool was mixed with diluent and the test strip was dipped into the tube followed by recording the results.

We conducted the PCR from the dried stool on filter paper as follows. The stool spot was cut into small pieces and placed into a microfuge tube, washed, and 150 µL of nuclease-free deionized water was added, followed by heat lysis at 100°C for 10 minutes. The lysate was used for multiplex PCR on a Thermo cycler C-1000 instrument (Bio-Rad) targeting *V*. *cholerae* O1-*rfb* and *ctxA* genes [25]. PCR products were analyzed on a 2% agarose gel using Gel Red (BioTium, USA) stain. To confirm the discordant results between RLDT and PCR, we used another set of primers targeting the gene of outer membrane protein (OmpW) [26].

Rapid LAMP-based Diagnostic Test assay was performed directly from fresh stool specimens using the RLDT kit as previously described [10]. In short, a stool sample was added to the sample processing tube containing lysis buffer, processed by heat lysis, and the lysate was added to the RLDT reaction strips, which were transferred to a battery-operated handheld reader for reading the result in 40 minutes. The RLDT strips contain all the reagents needed for amplification, already lyophilized, targeting O1 *rfb* and *ctxA* genes, and an assay inhibitor control. The positive or negative result was interpreted automatically by the reader [10].

### Cholera Detection From the Water Samples

From the cholera patients enrolled in this study, the first 50 patients who gave consent to collect drinking water from their households were chosen. The water samples were collected from either source water (eg, tap water) or stored water. After 400 mL of water samples were filtered through a 0.22 μm GV membrane (GVWP02500, Durapore, Merck Millipore, Ireland), the membrane was cut in half, and one half was used for RLDT directly [10]. The remaining half was placed in APW to enrich for overnight at 37°C, followed by culture on Taurocholate-Tellurite-Gelatin Agar (TTGA) and serotyping. For RLDT, the first half of the membrane was cut into small pieces and heat lysed using lysis buffer, followed by process as described for stool [10] and also tested from the enriched samples.

### Statistical Analysis

Cohen's kappa was used to describe the agreement between a pair of detection methods. GraphPad Prism7 was used for analysis and graphical presentation of data. Sensitivity and specificity of different diagnostic tests were calculated using the R version 4.5.1. Although RLDT detects both O1 and *ctxA* genes, since the latter alone could be confirmatory of cholera, the results shown in this analysis are based primarily on the positivity of *ctxA* gene for both RLDT and PCR, unless otherwise noted.

### Interpretation of Cohen's Kappa Test

Cohen's kappa is a reliable statistical measure used to assess both interrater and intrarater agreement. Like correlation coefficients, its values range from −1 to +1, where 0 reflects agreement expected by chance and 1 indicates perfect agreement between raters. Interpretation of kappa values is as follows: values ≤0 indicate no agreement; 0.01–0.20 suggest none to slight agreement; 0.21–0.40 indicate fair agreement; 0.41–0.60 represent moderate agreement; 0.61–0.80 indicate substantial agreement; and 0.81–1.00 reflect almost perfect agreement.

## RESULTS

### Clinical Characteristics of Study Participants

Clinical and demographic characteristics of all study participants are summarized in Table 1. Of the 210 suspected cholera patients, the majority were adults and had severe dehydration. Prior to the hospital visit, 35% patients had taken antibiotics. Patients with moderate or severe dehydration were treated with recommended antibiotics along with rehydration with oral rehydration solution (ORS) or intravenous fluid as required.

**Table 1.** Characteristics of the Patients Enrolled in the Study

Characteristics	Values (%)
No. of male patients	119 (57)
Median age in year (25th and 75th centiles)	30 (22 and 38)
Children, ≤18 y of age	38 (18)
Adult patients	172 (82)
Patients with severe dehydration	125 (60)
Patients with moderate dehydration	72 (34)
Patients with no dehydration	13 (6)
Antibiotic taken before coming to the study hospital	73 (35)

### Cholera RLDT Set Up and Training in Bangladesh

Rapid LAMP-based Diagnostic Test kits were shipped to the site. A team from Johns Hopkins University, visited the cholera reference laboratory, Dhaka, Bangladesh, to train the laboratory staff at icddr,b. In brief, a copy of the English test kit manual containing illustrations on the test procedure was provided along with a PowerPoint Presentation, followed by a hands-on training session on the first day. On the second day, the lab personnel performed the test under the observation of the trainers, and from the third day, they conducted the tests independently.

### Comparison of Cholera RLDT Assay With Other Assays

**Figure 1.**
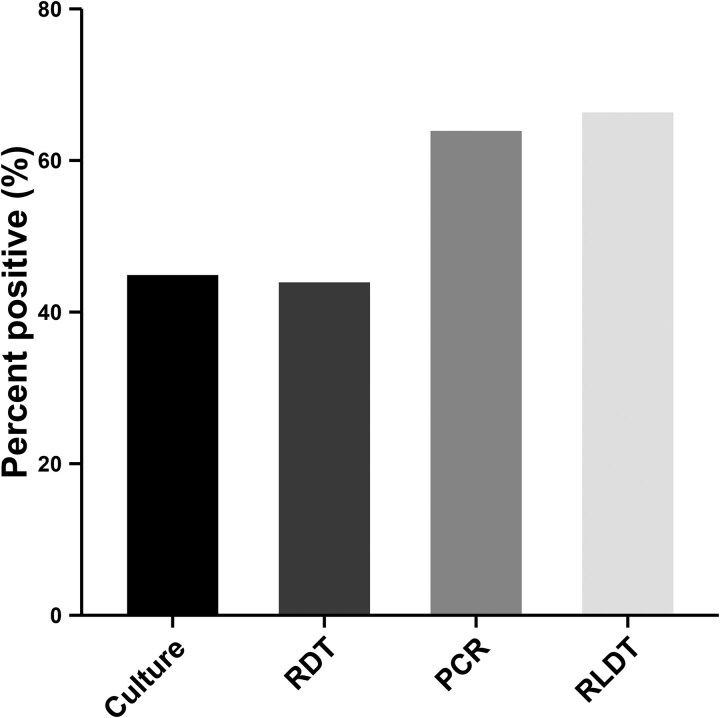
Histogram of the percentages of samples positive for cholera by each method of detection.

Out of 205 suspected cholera cases analyzed, 136 (66%) were positive for cholera by RLDT, 131 (64%) by PCR, 92 (45%) by direct or enriched culture, and 90 (44%) by RDT (Figure 1). Among the culture positives, 11 (11.96%) were O1 Ogawa, and the rest were O1 Inaba. Since 5 of the samples gave discrepant results between the 2 different PCR assays used and we are uncertain of the correct PCR results, we excluded these samples from the analysis.

### Similarities and Differences Between the Four Diagnostic Assays

**Figure 2.**
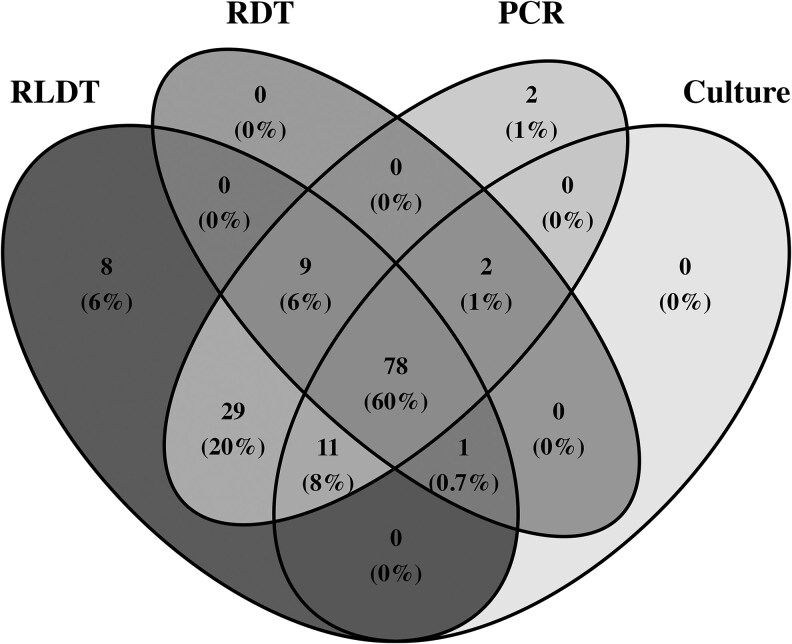
A Venn diagram that illustrates the overlap of the positive test results from 4 diagnostic methods.

Overall, 78 (60%) of the suspected cholera cases were positive by all 4 assays (Figure 2). A total of 92 stool samples were positive by culture, of which 89 were positive by direct culture, and an additional 3 samples were positive after enrichment in APW for overnight. Among these 92 culture positives, 90 were also positive by RLDT. Of the 2 samples that were negative, one was only positive for O1, and the other was negative for both genes by RLDT. Of the culture positives, 91 were positive by PCR, and only 81 were positive by the RDT. Rapid LAMP-based Diagnostic Test detected 46 (22.44%) and 48 (23.41%), and PCR detected 40 (19.51%) and 42 (20.49%) more cholera cases than culture and RDT, respectively. A total of 127 cases were positive by both the PCR and RLDT. Rapid LAMP-based Diagnostic Test detected an additional 8 cases, and PCR detected an additional 2 cases, which were negative by the other 3 assays. In RDT, 2 of the samples were positive for both O1 and O139.

### Pairwise Comparison Between the RLDT and Three Diagnostic Assays

**Figure 3.**
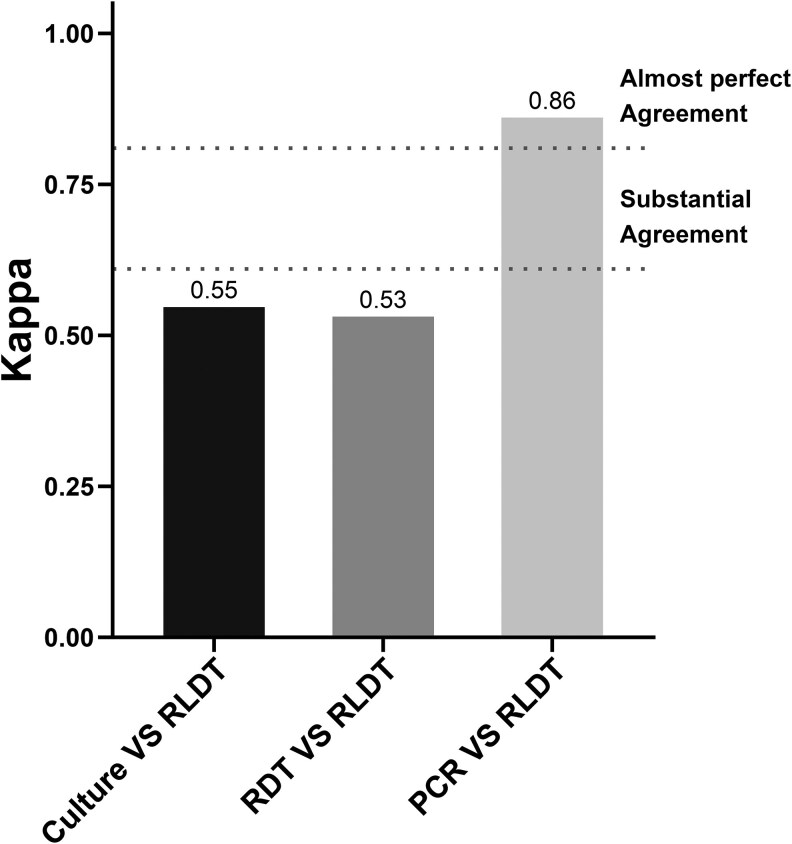
Pairwise comparison between RLDT, Culture, RDT, and PCR.

The Cohen's kappa test revealed almost perfect agreement between RLDT and PCR (kappa 0.86). The agreement between RLDT and RDT and RLDT and culture was similar, with a near substantial agreement of kappa 0.55 and kappa 0.53, respectively (Figure 3).

**Figure 4.**
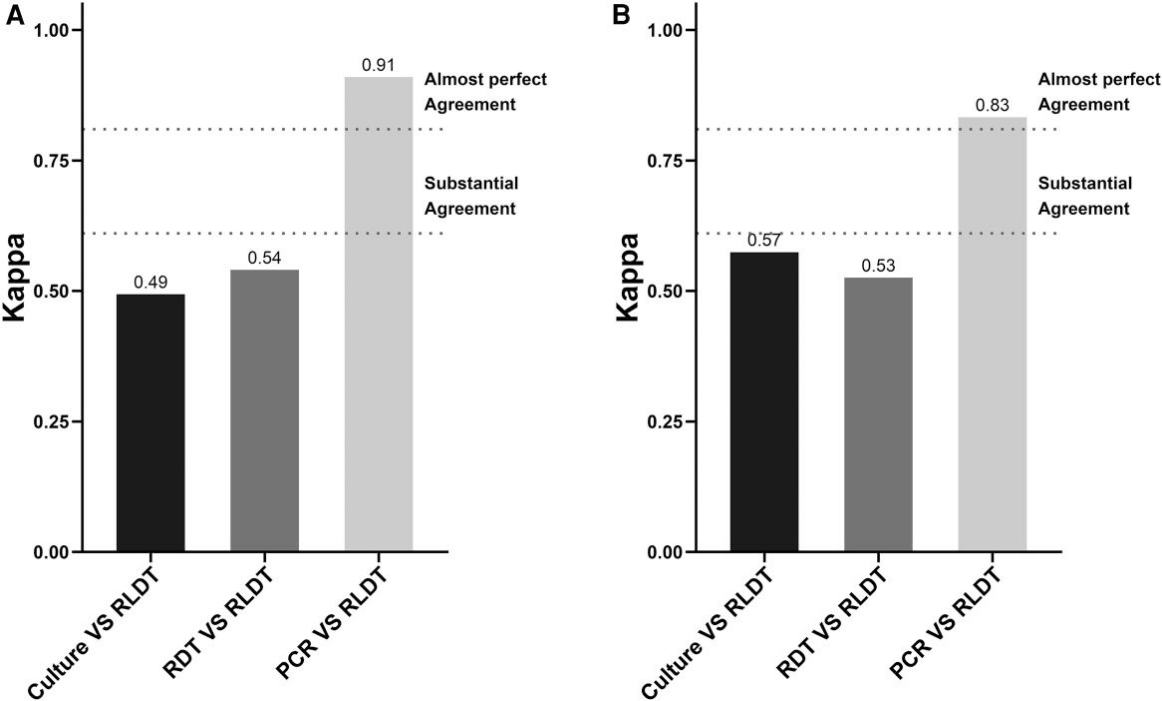
Pairwise comparison between the diagnostic assays among the patients based on prior antibiotic consumption. *A*, Illustrates the agreements between assays among patients who received antibiotics before seeking care at the icddr,b hospital, and (*B*) shows the agreements among patients who did not receive prior antibiotics.

We also analyzed the agreement between assays among the patients who received antibiotics before coming to the hospital and those who did not (Figure 4*A* and 4*B*). The agreement between RLDT and PCR increased to 0.91 among those with prior antibiotics, while it decreased to 0.83 among those who did not. The agreement between RLDT and culture was decreased to kappa 0.49 among those who had antibiotics, while the agreement between RLDT and RDT remained unchanged between the 2 groups.

Although none of the diagnostic assays used in this study could be considered the gold standard, and a comparison of RLDT with another assay as the gold standard may not be justified, we still analyzed the sensitivity, specificity, positive predictive value (PPV), and negative predictive value (NPV) of RLDT compared to other assays as the gold standard (Supplementary Table 1). Overall, the sensitivity of RLDT was over 90% across the comparison with all other assays, while the specificity widely varied (58.26%–97.73%) since RLDT and PCR are much more sensitive than culture and RDT. Considering *ctxA* as the target gene, compared to PCR, RLDT had a sensitivity of 96.95% and a specificity of 87.84%. Considering that when either O1 or *ctxA* is positive, the sample is positive for cholera, and the sensitivity and specificity of RLDT were 97.73% and 86.3%, respectively. The sensitivity of RLDT varied between 90.22% and 97.83% and 93.33% and 98.89% compared to culture and RDT, respectively.

### Evaluation of RLDT in Water Samples

A total of 50 drinking water samples were tested. After overnight enrichment in APW, 5 (10%) samples were positive by both the RDT and culture. While 2 (4%) were positive by the direct RLDT without enrichment, all 5 were positive by the RLDT when tested after enrichment. Of the positive samples, 1 was O1 Inaba, and the rest were O1 Ogawa; the serotypes matched between the *V. cholerae* isolates from clinical and water samples.

## DISCUSSION

This is the first evaluation of cholera RLDT in a low and middle-income (LMIC) country. This study demonstrated that lab staff members in a laboratory in LMICs can be trained in cholera RLDT, and the assay could be successfully implemented. We also showed that RLDT is highly sensitive and has substantial agreement with other assays.

As populations in LMICs increasingly coalesce in overcrowded mega-cities with poor sanitation, and global mobility accelerates coupled with more frequent extreme weather events, new and more virulent strains of *V. cholerae* are expected to spread more quickly and thus outbreaks are likely to rise in vulnerable regions [27–29]. This underscores the urgent need for stronger prevention measures and faster response strategies. Achieving this depends on having rapid, accurate diagnostic tools. Diagnosing cholera early at the onset of an outbreak, at the field level, should allow for a more timely response and mitigate the size, scope, and duration of the outbreak and subsequent spread of the illness. In this regard, as described before, culture and PCR are inadequate due to their complexity, being lengthy, requiring resources, and skilled personnel. Thus, timely diagnosis calls for RDTs. However, field and laboratory evaluations of the commercially available RDTs demonstrated variability in performance [11–18].

Rapid LAMP-based Diagnostic Test is a simple and rapid molecular test and has the potential to fill this void. In this study, the laboratory staff members were trained in RLDT in Bangladesh, where cholera is endemic, and the assay could be successfully implemented. We demonstrated that the sensitivity and specificity of RLDT were 97% and 88%, respectively, compared to PCR, with 90% agreement between these 2 assays. Thus, our study shows that while RLDT, in contrast to PCR, is simple, rapid (<1 hour), cold-chain, and almost electricity-free, requires minimum equipment, but on the other hand, has a similar sensitivity as PCR.

This study also demonstrated that RLDT was highly specific, with a sensitivity of 97% and 99% when compared to PCR and culture, respectively. In addition, RLDT could detect 24% more cholera than culture, of which 19% was confirmed by PCR. Like culture, the crystal VC RDT was also less sensitive than RLDT, and the latter detected 23% more cholera cases than the RDT, of which 20% were also positive by PCR. Other than early outbreak detection, the high sensitivity of the RLDT also has an advantage as a surveillance tool to estimate the real burden of cholera, as well as to estimate the efficacy of the oral cholera vaccine (OCV), since the shedding of *V. cholerae* in the stool sample could be reduced due to protection from the OCV.

Of note, in this study at the cholera reference laboratory, the RDT missed 11cholera cases, which were positive by the other 3 assays. As shown before, the accuracy of RDT compared to culture could be improved by enrichment in 1% APW for ∼6 hours; however, the sensitivity compared to PCR remained moderate [30, 31]. Notably, extending the RDT test incubation by 6 hours may impose an additional burden on the already strained health facilities at LMICs, as this may not align with the fixed working hours of health facility staff members.

As described before, the positivity of cholera by culture could be affected by prior antibiotic consumption [32]. Although RLDT is a molecular test, interestingly, the agreement between RLDT and PCR was higher among the prior antibiotic group, while, as expected, it was lower for the agreement between culture and RLDT. The agreement between RLDT and RDT was not affected by antibiotics, as reported before [32]. Antibiotic may have affected the PCR results, which needs to be further investigated.

Rapid LAMP-based Diagnostic Test could also detect cholera in drinking water, as found in this study; RLDT, after APW enrichment, was able to detect all 5 samples, which were positive by the enrichment RDT and culture. However, although RLDT showed an excellent limit of detection (LOD) in detecting cholera directly from spiked water samples in the laboratory [10], in this study, it failed to detect 3 of the 5 cholera-positive drinking water samples without enrichment.

Rapid LAMP-based Diagnostic Test detects both *ctxA* and O1*rfb*. In this evaluation, *ctxA* target was more sensitive than the O1*rfb*. In the future, only the *ctxA* target of the RLDT could be used to detect cholera, since *ctxA* alone could be confirmatory for cholera, and in case of the emergence of a new cholera serogroup (as happened with O139), the RLDT *ctxA* will still be able to detect the strain, which RDT will miss. Using only one target will reduce the cost, allowing more samples to be tested at a time. The RLDT *ctxA* positive samples could be tested later for O1 *rfb* to determine the serogroup if needed.

This study has limitations. In this study, for the convenience of the lab staff members, enrichment of water samples for overnight was used for the 3 assays. RLDT, however, had shown to have a LOD 10 colony forming unit (CFU) after 6 hours of enrichment (Chakraborty personal communication), which needs to be tested in the field in the future. Although the RLDT and PCR are more sensitive compared to culture, culture-based diagnostic methods remain crucial for downstream applications like antibiotic resistance monitoring, serotyping, and whole genome sequencing. To mitigate this challenge, since RLDT is rapid (<1 hour), the RLDT positive stool samples could be sent to the reference laboratory of the country for culture.

In conclusion, our results suggest that RLDT could be implemented in a reference laboratory in LMIC where cholera is endemic. Rapid LAMP-based Diagnostic Test was found to be highly specific and more sensitive compared to culture and RDT for the detection of cholera from stool. Rapid LAMP-based Diagnostic Test could also accurately detect cholera from drinking water samples. Rapid LAMP-based Diagnostic Test assures broader application as a culture-independent, simple, rapid, and cheap diagnostic test for cholera detection in resource-poor settings, where such a simple and field-adapted assay has a substantial impact on controlling cholera outbreaks or epidemic emergency situations. Testing of cholera RLDT in health care settings is underway.

## Supplementary Material

ofag292_Supplementary_Data

## Supplementary Data

Supplementary materials are available at *Open Forum Infectious Diseases* online. Consisting of data provided by the authors to benefit the reader, the posted materials are not copyedited and are the sole responsibility of the authors, so questions or comments should be addressed to the corresponding author.
