# Supplementary material for: Diagnostic Accuracy of a Simple and Rapid Diagnostic Test RLDT for the Detection of Cholera in Bangladesh
Source: Open Forum Infect Dis. 2026 May 14;13(5):ofag292. doi: 10.1093/ofid/ofag292 (PMC13218445; doi:10.1093/ofid/ofag292)
Supplement: ofag292_Supplementary_Data [file ofag292_supplementary_data.docx]

**Supplement Table 1. Performance of the RLDT compared with other diagnostic assays in the detection of cholera**

| Targets | Total Samples Screened | Prevalence by Gold Standard, N (%) | Prevalence by RLDT, N (%) | False Positive, N (%) | False Negative, N (%) | Sensitivity (%) | Specificity (%) | PPV (%) | NPV (%) | Accuracy (%) | Kappa |
| --- | --- | --- | --- | --- | --- | --- | --- | --- | --- | --- | --- |
| **RDT vs RLDT** |  |  |  |  |  |  |  |  |  |  |  |
| RDT O1 vs RLDT O1 | 205 | 90 (43.90) | 114 (55.61) | 29 (14.15) | 5 (2.44) | 94.44 | 74.78 | 74.56 | 94.51 | 83.41 | 0.6728 |
| RDT O1 vs RLDT ctxA | 205 | 90 (43.90) | 136 (66.34) | 48 (23.41) | 2 (0.98) | 97.78 | 58.26 | 64.71 | 97.1 | 75.61 | 0.5309 |
| RDT O1 vs RLDT O1+ctxA (at least one positive) | 205 | 90 (43.90) | 139 (67.80) | 50 (24.39) | 1 (0.49) | 98.89 | 56.52 | 64.03 | 98.48 | 75.12 | 0.5231 |
| **PCR vs RLDT** |  |  |  |  |  |  |  |  |  |  |  |
| PCR O1 vs RLDT O1 | 205 | 131 (63.90) | 114 (55.61) | 3 (1.46) | 20 (9.76) | 84.73 | 95.95 | 97.37 | 78.02 | 88.78 | 0.7684 |
| PCR ctxA vs RLDT ctxA | 205 | 131 (63.90) | 136 (66.34) | 9 (4.39) | 4 (1.95) | 96.95 | 87.84 | 93.38 | 94.2 | 93.66 | 0.8605 |
| PCR O1+ctxA (at least one positive) vs RLDT O1+ctxA (at least one positive) | 205 | 132 (64.39) | 139 (67.80) | 10 (4.88) | 3 (1.46) | 97.73 | 86.3 | 92.81 | 95.45 | 93.66 | 0.8587 |
| **Culture vs RLDT** |  |  |  |  |  |  |  |  |  |  |  |
| Culture O1 vs RLDT O1 | 205 | 92 (44.88) | 114 (55.61) | 30 (14.63) | 8 (3.90) | 91.3 | 73.45 | 73.68 | 91.21 | 81.46 | 0.6335 |
| Culture O1 vs RLDT ctxA | 205 | 92 (44.88) | 136 (66.34) | 46 (22.44) | 2 (0.98) | 97.83 | 59.29 | 66.18 | 97.1 | 76.59 | 0.5469 |
| Culture O1 vs RLDT O1+ctxA (at least one positive) | 205 | 92 (44.88) | 139 (67.80) | 48 (23.41) | 1 (0.49) | 98.91 | 57.52 | 65.47 | 98.48 | 76.1 | 0.5388 |
| Likelihood ratio |  | **Positive likelihood ratio** | **Negative likelihood ratio** |  |  |  |  |  |  |  |  |
| RLDT vs PCR |  | 28.8 | 0.13 |  |  |  |  |  |  |  |  |
| RLDT vs Culture |  | 27.3 | 0.42 |  |  |  |  |  |  |  |  |
| RLDT vs RDT |  | 26.2 | 0.43 |  |  |  |  |  |  |  |  |
|  |  |  |  |  |  |  |  |  |  |  |  |
|  |  |  |  |  |  |  |  |  |  |  |  |
|  |  | sensitivity/(1-specificity) | specificity/(1-sensitivity) |  |  |  |  |  |  |  |  |
